# Commercial Off-the-Shelf Components (COTS) in Realizing Miniature Implantable Wireless Medical Devices: A Review

**DOI:** 10.3390/s22103635

**Published:** 2022-05-10

**Authors:** Sadeque Reza Khan, Andrew J. Mugisha, Andreas Tsiamis, Srinjoy Mitra

**Affiliations:** Institute of Integrated Micro and Nano Systems, School of Engineering, University of Edinburgh, Edinburgh EH9 3FF, UK; s1648757@ed.ac.uk (A.J.M.); a.tsiamis@ed.ac.uk (A.T.); srinjoy.mitra@ed.ac.uk (S.M.)

**Keywords:** commercial off-the-shelf (COTS), encapsulation, implantable wireless medical devices (IWMDs), telemedicine, wireless body area networks (WBAN), wireless power transfer (WPT)

## Abstract

Over the past decade, there has been exponential growth in the per capita rate of medical patients around the world, and this is significantly straining the resources of healthcare institutes. Therefore, the reliance on smart commercial off-the-shelf (COTS) implantable wireless medical devices (IWMDs) is increasing among healthcare institutions to provide routine medical services, such as monitoring patients’ physiological signals and the remote delivery of therapeutic drugs. These smart COTS IWMDs reduce the necessity of recurring visits of patients to healthcare institutions and also mitigate physical contact, which can minimize the possibility of any potential spread of contagious diseases. Furthermore, the devices provide patients with the benefit of recuperating in familiar surroundings. As such, low-cost, ubiquitous COTS IWMDs have engendered the proliferation of telemedicine in healthcare to provide routine medical services. In this paper, a review work on COTS IWMDs is presented at a macro level to discuss the history of IWMDs, different networked COTS IWMDs, health and safety regulations of COTS IWMDs and the importance of organized procurement. Furthermore, we discuss the basic building blocks of IWMDs and how COTS components can contribute to build these blocks over widely researched custom-built application-specific integrated circuits.

## 1. Introduction

Telemedicine paves the way for routine healthcare inspection and facilities the provision of care to patients from remote locations. Wearable and in-body medical devices are increasingly used to provide telemedicine services for routine diagnostic and drug delivery services for elderly, vulnerable and disabled patients. Such devices minimize patients’ commute frequency to healthcare centers and ensure that patients can be monitored either for regular checkup or any post-surgery recovery in their familiar surroundings. Therefore, telemedicine services can prevent healthcare institutions from being overwhelmed and overcrowded in regular conditions or any pandemic situation. The effective functioning of a telemedicine ecosystem depends on four key sectors [1], which are consumers, innovators, investors and regulators. Consumers include patients, physicians and hospitals. Innovators are mainly the tech start-ups, incubators and global corporations. Furthermore, investors are venture capitalists, non-profit bodies or banks. The regulator sector includes organizations, such as the Food and Drug Administration (FDA), the Federal Communication Commission (FCC) and the World Health Organization (WHO). The telemedicine ecosystem has evolved significantly as the demand for remote access to e-health services grows [2]. Additionally, point of care (POC) represents a key part of the improvement of telemedicine and healthcare management. It is crucial to manage a disease, in terms of progression and monitoring evaluation, according to the patient’s profile [3]. Wireless and implantable POC devices can facilitate this by regular monitoring and providing quick acquisition of test results so that the patient can be treated in the right direction at the earliest [4], which can improve telemedicine significantly.

One of the major influences on the advancement of telemedicine is the development cycle of different implantable devices. Compared to wearable medical sensors, implantable devices take longer to develop and reach the customer. Implantable devices are generally classified as tethered (also known as wired) and wireless implants. Implantable wireless medical devices (IWMDs) are a more suitable choice in lieu of wired implants due to the high risk of infections caused by tethered implants [5]. Furthermore, tethered implants are prone to bio-signal artifacts caused by patient movement [6]. A patient with an IWMD is less likely to develop a catheterized infection or cause motion-triggered bio-signal artifacts compared to a tethered implant. All these features have increased the acceptance of IWMDs among patients over the past decade.

In the past two decades, custom-built application-specific integrated circuit (ASIC) remains the primary choice for the development of IWMDs [5,6,7,8,9]. Most of these chips intend to develop RF-integrated circuits (RFIC) for wireless connectivity within miniature implantable medical devices. However, custom designed ASIC-based IWMDs tend to be encumbered with challenges such as time consuming development cycles, repeatability and reliability challenges. Furthermore, high costs associated with software support and hardware prototyping is another major issue associated with custom-built ASIC-based IWMDs. Alternatively, recent advancements in micro- and nano-scale semiconductor technologies have been instrumental in the rapid development of low-cost and miniaturized commercial off-the-shelf (COTS)-enabled IWMDs [10,11]. Driven by Moore’s law [12], the computational performance, efficiency and precision of COTS components have grown exponentially, as their associated manufacturing costs have fallen in proportion. The ubiquity of low-cost COTS components used in developing IWMDs [13] benefits from economies of scale derived from short development cycles, low development costs, high yield from mass manufacturing, robust and replaceable devices and manufacturers’ support from hardware and software development tools.

In this review, Section 2 describes the brief history of wireless implants, how IWMDs contribute to the healthcare network and safety aspects of the IWMDS. Section 3 explains the basic building blocks of COTS-enabled IWMDs and their operation principle. Section 4 compiles recent publications on RF transceivers for COTS-enabled IWMDs and offers a brief suggestion regarding the usage of these devices, followed by conclusions.

## 2. Macro Purview of COTS-Enabled IWMDs

### 2.1. History of Wireless Implants

IWMDs date as early as 1948 when Fuller et al. presented an innovative device which performed sample recordings of human carotid pulses and human heart rate during a period of apnea, measured human finger pulse and performed a canine pneumogram [14]. This research led to multiple historical advancements in custom IWMDs, illustrated in Table 1.

With advances in manufacturing processes and integration techniques, the commercialization of low-cost miniaturized COTS-based IWMDs emerged circa 1966. Table 2 illustrates a historical timeline of commercial COTS-enabled IWMDs. Technological advances have engendered an era of smart networked IWMDs.

### 2.2. Role of IWMDs in Healthcare Networks

**Figure 1:**
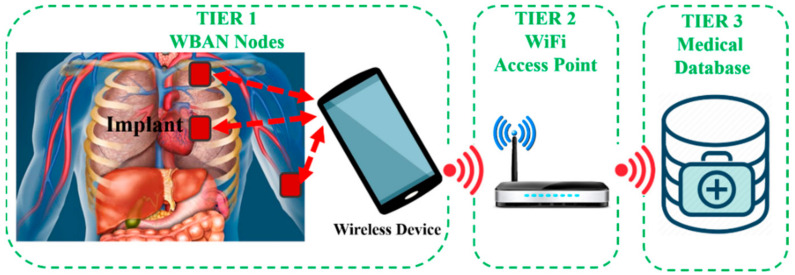
Contribution of IWMDs in a WBAN-based healthcare network.

The proliferation of COTS-enabled hardware platforms across healthcare networks accelerated the convergence of information technology in the medical industry [25]. Commercial wireless biosensors have emerged significantly in the past decade to fulfil the demand for wireless medical equipment for patient monitoring and personal healthcare [26]. Figure 1 illustrates the contribution of COTS-enabled IWMDs in a tiered healthcare network. Tier-1 comprises wireless body area network (WBAN) nodes. The WBAN nodes represent both wearables and IWMDs, which collect and process bio-signals prior to transferring the biotelemetry data to a secured WBAN gateway. The primary aim of WBAN is to ensure continuous monitoring of the patient’s vital parameters while giving them the freedom of movement, which results in the enhancement of the quality of healthcare [27]. Furthermore, Tier-2 bridges WBAN nodes to medical databases via access points such as Wi-Fi. Finally, Tier-3 represents a medical server infrastructure from which healthcare providers can access patient records. IWMDs play a significant role in the improvement of the overall efficiency of the healthcare network by allowing the data management of patients through a single standard wireless device (e.g., smart phone or a hand-held antenna), as shown in Figure 1 [26].

### 2.3. Regulatory Guidelines for Medical Devices

The World Health Organization (WHO) provides the global medical device nomenclature (GMDN) online repository for certified implantable medical devices [28]. Furthermore, the Food and Drug Administration (FDA) is responsible for publishing federal guidelines on medical device safety. Additionally, regulatory requirements and policies on operating wireless medical devices are coordinated by the Federal Communications Commission (FCC) [29]. These are the primary regulatory organizations to recognize a medical device to be certified for human use. Due to their maturity and established development cycle, COTS-enabled IWMDs have a significant advantage in acquiring regulatory approvals. It should be noted that substantial resources have also been assigned to the procurement of COTS components. The U.S. Air Force Materiel Command published a COTS procurement document which provides guidelines for the identification, selection, acquisition, logistics support and testing of COTS equipment [30]. Original equipment manufacturers (OEMs), such as Texas Instruments (TI), have product portfolios on advanced and sophisticated COTS components for medical, avionics, defense and space electronics industries, as illustrated in [31].

### 2.4. Health and Safety

#### 2.4.1. Effects of Electromagnetic (EM) Energy on Human Body

COTS-enabled IWMDs must adhere to guidelines on health and safety with respect to EM energy exposure and absorption [32,33,34,35,36]. Tissue safety regulations for IWMDs is measured with near-field specific absorption rate (SAR) and far-field power density (PD). However, SAR is the most commonly used metric [35] to quantify the tissue safety against any IWMD. Widely accepted IEEE standards for SAR levels are FCC-approved 1.6 W/kg averaged over 1 g of tissue and European Union-approved 2 W/kg averaged over 10 g of tissue [37].

#### 2.4.2. Encapsulation

**Figure 2:**
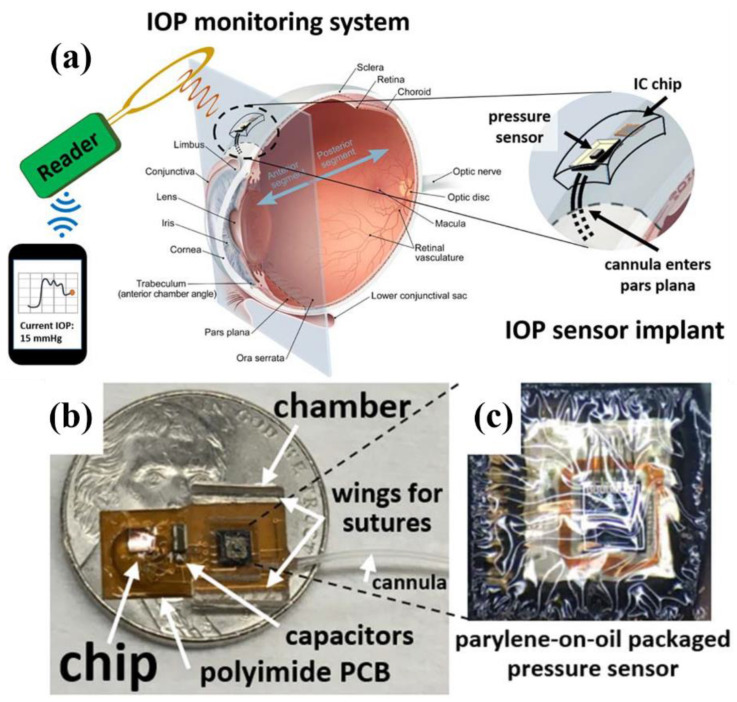
Example of an encapsulated wireless implant [38]. (**a**) Continuous IOP monitoring system. (**b**) Overall implemented system. (**c**) Proposed system encapsulated in parylene-on-oil.

Robust encapsulation ensures the safe and stable operation of IWMDs inside the human body. Commercially available organic polymers such as polyimide, parylene and polydimethylsiloxane (PDMS) are frequently used to encapsulate implantable devices. In Figure 2, an IWMD intraocular pressure (IOP) monitoring system is presented [38], where the overall system is encapsulated using parylene-on-oil. A COTS ST-Microelectronics LPS25H pressure sensor [39] is used for IOP monitoring.

#### 2.4.3. Failure Due to Multiple Medical Steam Sterilization

Medical steam sterilizers (MSSs) utilize extremely fast cycles of temperature, humidity and pressure to sterilize medical instruments, which represents an exceptionally harsh environment for electronics components [40]. Therefore, it is necessary to study the failure mechanisms of plastic-encapsulated modules such as IWMDs and determine the level of deterioration of encapsulant adhesion over multiple sterilization cycles. This is an important step to confirm the purity and suitability of the long-term usage of implantable medical devices. This work demonstrates a COTS-enabled steam sterilizable plastic-encapsulated wireless sensor module (WSM) used to study the root causes of failures by exposing it to multiple steam sterilization cycles. The application was reliable for 100 cycles at high-rate temperature, pressure and humidity cycles at 134 °C, 3 bar and 100% RH, respectively. Although fractures, encapsulant delamination and electrical failure were some of the stress factors observed on the WSM after 100 cycles, the application has promising attributes. The study in [40] also shows that good reliability can be achieved at 3 mm on HTFR4 Epoxy-glass PCB and FP4460 Epoxy encapsulant. Furthermore, the failure due to temperature cycling is disproportional, and larger components are more exposed to the thermomechanical stress. The humidity storage and humidity cycling produce low levels of electrical failures but higher levels of adhesion loss due to the moisture ingress to the adhesive interface.

## 3. Essential Building Blocks of COTS-Enabled IWMDs

**Figure 3:**
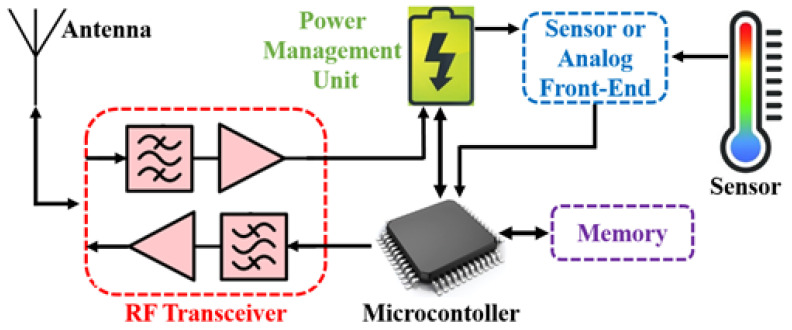
Essential blocks for a COTS-enabled IWMD.

It is preferable to use low-profile and compact form factor COTS due to the invasive nature of IWMDs to minimize patient discomfort. Generally, the physical dimensions of antennas and energy storage devices, such as rechargeable batteries, dominate the form factor in an IWMD. It is necessary to accurately define the specification of an IWMD’s essential blocks to achieve the optimal form factor without compromising functionality. In this section, essential blocks for a COTS-enabled IWMD are described, as shown in Figure 3. It includes an antenna unit to communicate with the wireless device, a radio frequency (RF) transceiver to process the input/output signal, a power management unit to rectify and regulate the necessary power, a microcontroller as a data acquisition and processing unit, a sensor, and any required sensor or analog front-end to collect necessary bio-signals and memory device to store data (for delayed transmission, offline data download, etc.).

### 3.1. Essential Blocks Description

#### 3.1.1. Antenna

**Figure 4:**
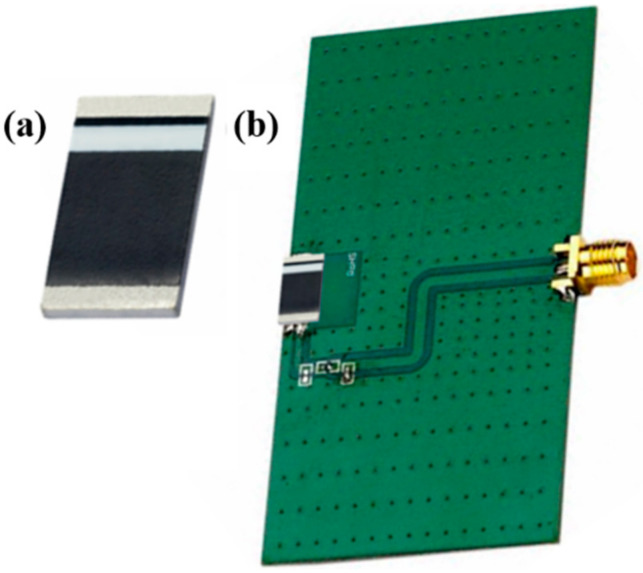
COTS Taoglas 868 MHz ceramic chip antenna: (**a**) 5 mm × 3 mm × 0.5 mm form factor; (**b**) 80 mm × 40 mm × 0.8 mm evaluation board [41].

Figure 4 shows the dielectric resonant antenna (DRA) packaged in a chip form, which is increasingly used in the development of miniature COTS components for wireless applications.

Table 3 lists the DRAs compatible with COTS to be integrated with wireless system on chip (SoC) modules. DRA gain and efficiency are influenced by the IWMD’s ground plane dimensions. The evaluation board demonstrated in Figure 4 has a ground plane dimension represented as:

(1) $$D_{GND}=\frac{\lambda_{0}}{4}\times \frac{\lambda_{0}}{8}$$

where *λ*_0_/4 is the free space quarter wavelength at the frequency of the antenna. Reducing the size of the ground plane to be accommodated in the miniaturized IWMD proportionally degrades the gain and efficiency of the antenna.

#### 3.1.2. RF Transceiver

The high power consumption of RF transceivers is a major concern for battery-operated IWMDs. To conserve battery life, the transceiver section is usually disabled or forced into sleep mode until wireless connectivity is required.

In Table 4, the battery-operated COTS wireless devices mentioned in [47,48] utilize sleep mode functionality to conserve battery life while the NXP COTS [49] ultra-high frequency (UHF) radio frequency identification (RFID) chip remains off until powered by an RFID reader. In Table 4, the Ucode7 is significantly low power compared to ZL70101 and CC2541, as it is a battery-free RFID chip. However, it has lower operating distance compared to other two options. Furthermore, Ucode7 is significantly smaller than ZL70101 and CC2541, with size being an important parameter for IWMDs. It is also necessary to consider the frequency as it plays a key role in maintaining SAR limit.

**Figure 5:**
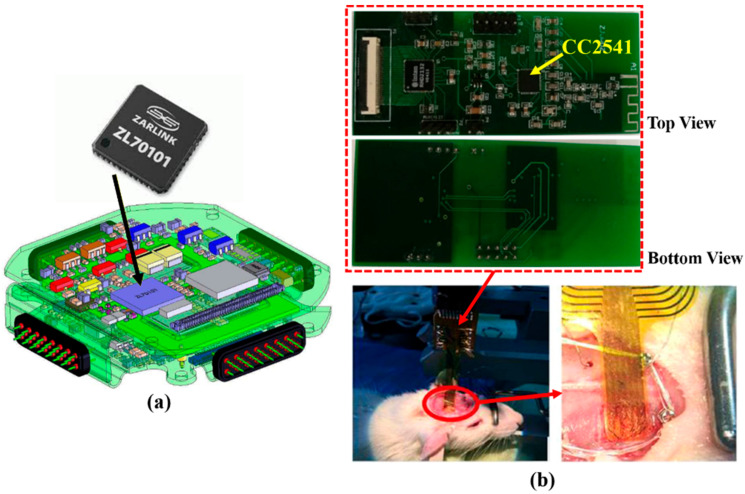
(**a**) The COTS Zarlink ZL70101 RF transceiver by Microsemi INC [55] used in wireless multi-channel acquisition system for generic interface with neurons (WIMAGINE) [47]. (**b**) Implantable flexible ECoG system using CC2541 tested on the left hemisphere of the brain of a Sprague–Dawley rat [22].

Figure 5a shows the ZL770101 chip used as a wireless data link module in a WIMAGINE neurological signal capture device. The basic architecture of WIMAGINE includes 64 platinum electrodes of 4.5 mm pitch, two CINESIC32 ASIC and an MSP430 ultra-low-power microcontroller [47]. The electronic environment around the CINESIC32 ASICs is implemented using COTS components.

Figure 5b shows an IWMD electrocorticography (ECoG) system built with COTS CC2541 [22]. The proposed system was tested on the left hemisphere of the brain of a rat, and based on the recording results and signal processing, it is possible to map out the ECoG signals over the measured area to locate the exact epilepsy lesions.

In [54], a multilayer tissue model of the neck and skin is verified by using NXP UCODE 7 COTS chip.

#### 3.1.3. Power Management Unit

**Figure 6:**
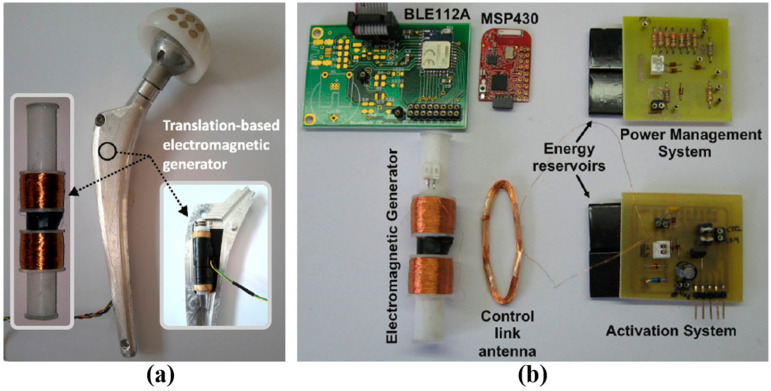
(**a**) Smart hip prosthesis prototype. (**b**) Modules and subsystems used to characterize and validate PMU performance [56].

The power management unit (PMU) is an important part in an IWMD as it can supply and maintain the necessary power required for optimal operation. A PMU generally consists of a rectifier, regulators, power monitoring circuit and safety unit. The use of a COTS PMU is demonstrated in a smart hip prosthesis comprising multiple energy harvesting systems [56]. In Figure 6, the power management system or PMU consists of a COTS MAXIM Integrated^TM^ MAX6777 low-power battery monitoring IC to track the output power of micro-power generators or energy harvesters for smart hip prostheses. There is also wireless power transfer (WPT) working as an alternative to the energy harvester. The power switches are implemented with COTS MOSFET transistors. Furthermore, the isolation between two power sources is accomplished by COTS Schottky diodes. Voltage regulation is conducted by an ultra-low-power and low-dropout MAXIM Integrated^TM^ MAX1963A voltage regulator. COTS Callergy CLG04P040F17 supercapacitors of 40 mF are used as energy reservoirs. In this work, the manufactured PCBs are used to test the system and subject to be miniaturized to fit in the implant.

In [57], an IWMD neuromodulation implant (bionode) is built using COTS components. To supply the required voltage to the bionode, a Texas Instruments (TI) TI7660 boost converter is used for positive output, and an inverted version of the same IC is used for negative output. Furthermore, the proposed system uses two Linear Technology LTC4071 chips as PMUs.

#### 3.1.4. Microcontroller Unit

The microcontroller unit (MCU) is an essential building block for most COTS-enabled IWMDs. Since processing speed and volume of the data are the key factors determining the higher power consumption, MCUs should be carefully chosen to match the requirement of the signal being sensed. Low clock rates (<10 kHz) are often sufficient for processing bio-signals in the range of a few Hz to 500 Hz. It should be noted that apart from the action potentials generated in a neuron, most bio-signals fall within this frequency range. It is also essential to factor in the MCU power consumption in sleep state (or deep sleep), since bio-signals rarely need continuous monitoring. As most physiological signals vary at a very low frequency, it is likely that the IWMDs can often be kept in a sleep state. A COTS-based MCU-enabled telemetry system for brain glucose measurements is presented in [58]. The application uses a COTS Microchip-PIC12F683 MCU [59].

**Figure 7:**
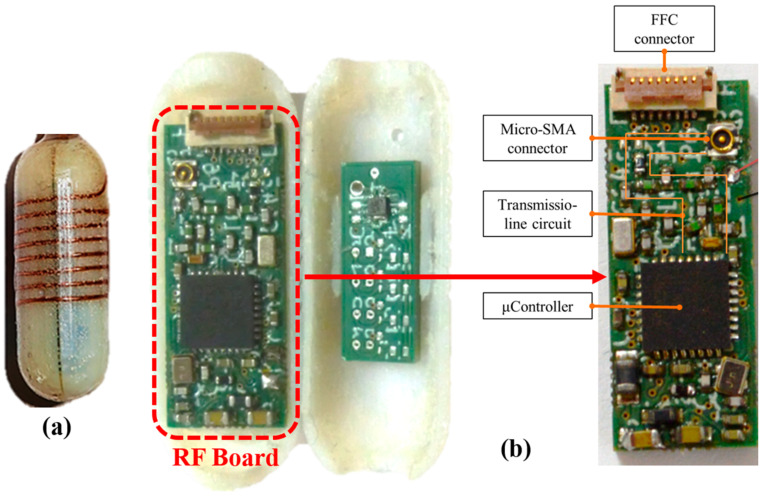
(**a**) Ingestible wireless capsule. (**b**) Position of the RF and thermistor PCB boards inside capsule [23].

In [23], a wireless ingestible capsule is presented with a telemetry module and a conformal helical antenna, as shown in Figure 7a. The size of the capsule is 30 mm in length and 10 mm in diameter. Figure 7b shows the position of the radio frequency (RF) and thermistor board inside the capsule; the RF board includes a COTS TI ARM Cortex-M3-based wireless microcontroller combined with an ultra-low-power RF transceiver (CC1310).

In [57], a closed-loop neuromodulation implant is presented with only COTS components. This device can record and transmit up to four channels of biopotential data while simultaneously providing biphasic constant current stimulation. In this work, a Nordic semiconductor nRF51822 Bluetooth low-energy microcontroller is used to process the electrode recording data amplified by a front-end amplifier.

#### 3.1.5. Analog Front-End (AFE) and Sensors

The AFE’s primary functions are to power up, detect and process low-frequency signals from external sensors. Comprehensive AFE functions include sensor biasing, multiplexing of sensor array output signals, linearization, amplification, offset control and temperature compensation [60,61,62]. Signals from external sensors are detected, captured and formatted by the AFE. To conserve energy, external sensors are usually deactivated, placed in sleep mode or a hibernation state until an external stimulus from an AFE activates the sensors.

**Figure 8:**
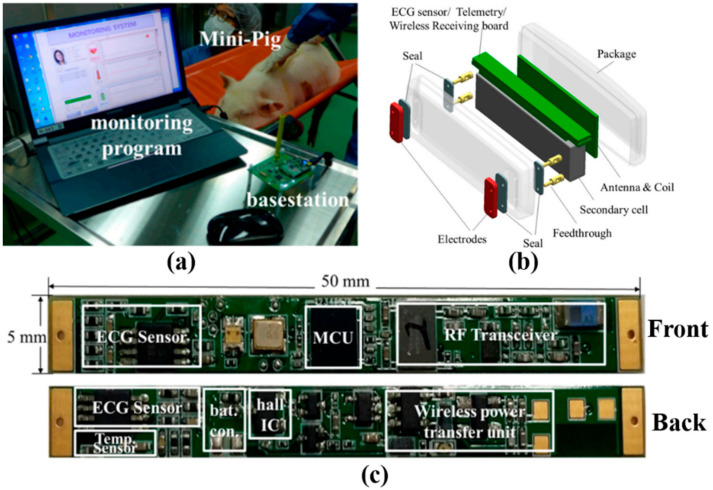
Implantable ECG monitoring system. (**a**) Experimental setup with the system implanted into subcutaneous fat in a mini-pig. (**b**) Overall system configuration. (**c**) PCB of the proposed system [63].

In [63], a wireless implantable ECG monitoring system is proposed and tested inside a mini-pig, as show in Figure 8a. Furthermore, Figure 8b,c shows the configuration of the implantable device and a two-sided view of the PCB, respectively. In this work, a COTS TI INA333 instrumentation amplifier is used to amplify the ECG differential signal.

Ref. [64] presents a wireless electrophysiological recordings and optogenetic stimulation platform where an Instant Technologies COTS RHD2132 is used, which includes a fully integrated electrophysiology amplifier with an on-chip 16-bit analog-to-digital converter (ADC). In [57], the AFE features high differential input impedance, low noise, a high common mode rejection ratio (CMRR) and sufficient gain and bandwidth. It includes two parallel dual-ended AFEs where each AFE consists of passive front-end filtering followed by two gain stages. The first and second stages are built with a COTS TI INA333 instrumentation amplifier and a TI OPA2313 general purpose opamp, respectively.

**Figure 9:**
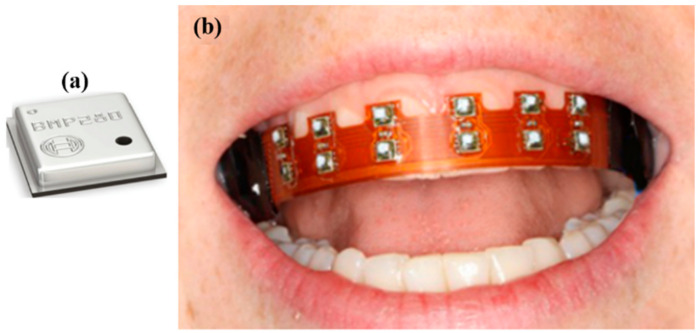
(**a**) A 2 mm × 2 mm× 0.95 mm BMP 280 digital barometric pressure sensor. (**b**) Implant application for wireless in situ measurement of lip pressure using BMP280 [68].

Additionally, there are different sensors used in IWMDs including ECG, EEG, electromyogram (EMG), bioimpedance analyzers, etc. IWMDs with COTS-based sensors are presented in [57,65,66,67,68]. Figure 9 illustrates an example in the form of a COTS digital barometric pressure sensor used to measure lip pressure.

In [38], a COTS STMicroelectronics digital output pressure sensor LPS25H [39] is used with internal temperature calibration. It is A 2.5 mm × 2.5 mm × 0.8 mm sized low-power sensor which can achieve a pressure resolution of 0.08 mmHg in the lowest power mode. Ref. [23] includes four COTS TDK Group negative temperature coefficient (NTC) thermistors B57540G1 used to monitor the temperature inside gastrointestinal (GI) tracts for capsule endoscopy application.

### 3.2. Consideration of the Number of COTS Components in IWMDs

**Figure 10:**
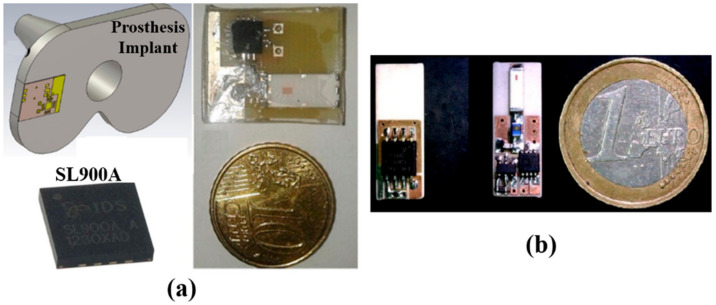
(**a**) Single COTS-based SoC IWMD implant [71] demonstrating fabricated IWMD including antenna and SL900A chip [69]. (**b**) Demonstration of LORAWAN signal for IWMD built with multiple COTS [72].

Compared to a completely custom-built IWMD, in COTS-enabled IWMDs, the device can be built with single or multiple COTS. A hybrid IWMD with both custom-built chip and COTS components is present in the literature. The SL900A SoC manufactured by Austria Microsystems AG (AMS) [69] is a COTS UHF-RFID sensory tag that can be implemented as a single chip solution for an IWMD. The AMS SL900A chip requires only an external antenna with the required matching elements. The 5 mm × 5 mm × 0.9 mm SL900A is remotely activated by a UHF RFID reader and can be operated as a battery-free IWMD. Figure 10a illustrates the implementation of the SL900A as part of an IWMD inserted in a prosthetic implant. Furthermore, Ref. [70] presented a wireless biofuel cell monitoring system using SL900A tested in vivo in a 12–16-week-old female Sprague–Dawley rat.

A battery-operated subcutaneous implantable device is presented in [73]. The implantable application includes a single Simblee™ Bluetooth^®^ smart module RFD77101, which is a 10 mm × 7 mm × 2.2 mm COTS SoC module with an integrated antenna [74]. The implant harvests solar energy ex vivo through 3 mm of porcine bio-tissue to replenish a 7 mAh rechargeable battery. The rechargeable battery powers a temperature sensor inside the RFD77101. In [24], a single Axzon Magnus S3 passive RFID chip is used with an antenna to wirelessly monitor local deep infections in orthopedic bone plates.

Although IWMDs are commonly assembled with multiple COTS components, in [75], the authors demonstrated a hermetically sealed wireless implantable neural interface realized using multiple COTS devices. The application consists of four key COTS system blocks for recording, stimulating, wireless telemetry and a power supply. Table 5 is a BoM for the implantable neural interface.

In [72], a low data rate transmission method is demonstrated for devices implanted in the body using backscattered long range (LoRa) signals, as shown in Figure 10b. The proof of concept includes analog devices ADG902 and LTC6907 used as the CMOS switch and oscillator, respectively. It also includes a COTS Semtech SX1278 LoRa transceiver.

A hybrid COTS–custom implantable wireless neuroprosthetic is presented in [76]. The application’s neuroprosthetic implantable device is custom designed and utilizes a COTS USRP B210 software defined radio (SDR) for telemetry.

### 3.3. Considerations of Power Supplies for IWMDs

Selection of the power supply for an IWMD is one of the most challenging decisions to make. The transceiver section in a battery-operated and compact wireless device consumes the largest portion of the application’s current. To maximize battery life, the transceiver block is enabled only when wireless connectivity is required, i.e., duty cycled to minimize current consumption. Power supply requirements for a miniaturized IWMD impact the device’s form factor. This also influences how long the device can operate for and the distance over which a robust wireless link for communication and power transfer can be maintained. To achieve prolonged use, rechargeable energy devices are the most demanding power source choice for battery-operated miniaturized IWMDs. This section describes some important parameters for IWMD power sourcing.

#### 3.3.1. Rechargeable Batteries for IWMD

**Figure 11:**
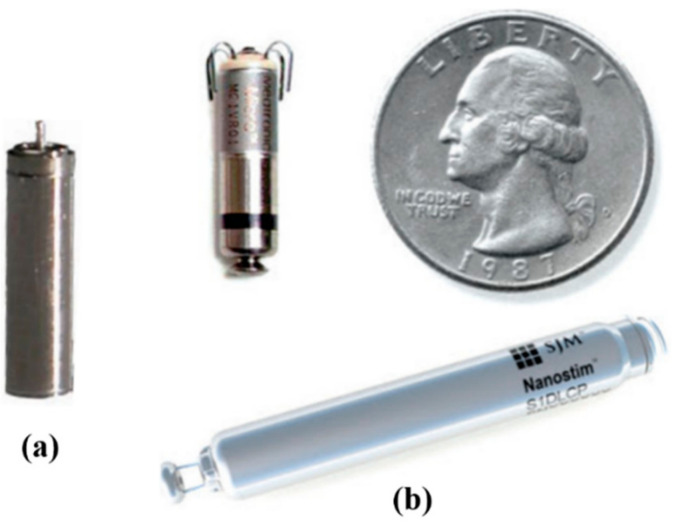
(**a**) A COTS 3.6 V 3 mAh QL0003B miniature rechargeable Li-ion battery used in pacemakers. The radius and height of the battery are 2.9 mm and 11.9 mm, respectively [81]. (**b**) State-of-the-art pacemakers: top left is the Micra and bottom right is the Nanostim [82].

Contemporary cardiac pacemakers rely on rechargeable batteries to operate continuously over several years. Innovative designs for pacemaker rechargeable batteries using lithium/iodine cells technology were presented in [77]. The authors reported rechargeable batteries with a volumetric energy density of 1.0 Wh/cm^3^ and an energy density of 270 mWh/g. Novel rechargeable batteries with a capacity of 3 mAh, a volume less than 0.1 cm^3^ and 3000 recharge cycles have been realized for potential use in IWMD [78]. A rechargeable COTS Li-ion battery QL0003 developed by Quallion LCC [79] was presented in [80] in which the authors demonstrated that the QL0003 could potentially be operated in an IWMD for up to 10 years on a single daily recharge. Figure 11a shows an example of QL0003B rechargeable batteries generally used in pacemakers.

#### 3.3.2. Regular Batteries for IWMD

Regular or non-rechargeable battery-powered IWMDs operate over a wider distance in comparison to their battery-free IWMD complements. A battery’s capacity (mAh) impacts an IWMD’s physical volume (form factor) and operational longevity. Pacemakers are the most notable battery-operated IWMDs. In [82], two commercially available pacemakers by St. Jude Medical are presented, the Micra transcatheter pacing system (Medtronic) and the Nanostim leadless cardiac pacemaker. The Micra and Nanostim, shown in Figure 11b, were meticulously evaluated for ventricular sensing, pacing and rate responsiveness. Both devices exhibited remarkable safety and efficacy results as an alternative to transvenous pacemakers.

#### 3.3.3. Battery-Free or Wirelessly Powered Transfer

Battery-free IWMDs are activated when in proximity to an energy source and thus are less likely to require internal energy storage devices. This type of device is called a passive mode device. The advantages of battery-free IWMDs are reduced form factors and absolutely no current consumption in the absence of external excitation. In contrast, battery-operated IWMDs continue to draw current in the nanoamps to microamps range when the device’s wireless transceiver is inactive. Examples of battery-free IWMDs realized using COTS UHF RFID devices were demonstrated in [83,84,85]. In comparison to battery-operated devices, the operating distance for battery-free wireless charging and telemetry are significantly shorter. Battery-free IWMDs have receive sensitivity of −7 dBm to −10 dBm whereas battery-operated IWMDs’ receive sensitivity ranges from −60 dBm to −90 dBm. UHF RFID battery-free IWMDs can be operated as battery-assisted passives (BAPs) [86,87] with −20 dBm receive sensitivity.

**Figure 12:**
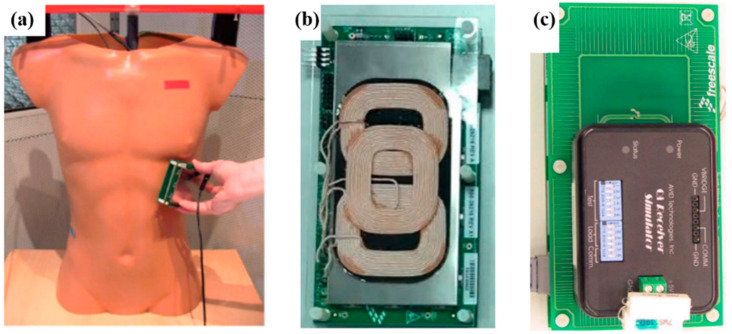
(**a**) EM interference test using a Qi-A13-Board. Test performed on torso phantom with CIED implant [93]. (**b**) NXP Qi-A13-Board [95]. (**c**) Avid Technologies Qi receiver stimulator [93].

Furthermore, wireless power transfer (WPT) technology has increasingly become the choice for powering battery-free and battery-operated wireless devices [88,89]. In WPT, time-varying EM energy from a source or transmitter propagates across free space and bio-tissue to replenish and/or power up an IWMD. In [90,91,92], the authors demonstrated how WPT sources are optimized to maximize power transfer efficiency (PTE) across free space and multiple layers of bio-tissue (skin, fat, muscle and bones) of varying physical dimensions. The wireless power consortium (WPC) is a collaborative group that develops standards for worldwide compatibility of wireless chargers which uses inductive coupling for power transfer. The WPC’s Qi specification is the world’s de facto wireless charging standard for small personal electronics. In [93], the health and safety considerations are presented for Qi-enabled devices. The authors also assessed the potential EM interference of a COTS-based NXP Qi-A13-enabled device as shown in Figure 12, when operated in the presence of active cardiovascular implantable electronic devices (CIEDs) with wireless power sources [94]. The authors also investigated whether permanent pacemakers (PPMs) and implantable cardioverter defibrillators (ICDs) were susceptible to EM fields generated by an active Qi-A13 device. The Avid Technologies Qi WPT receiver stimulator is used inside the torso, as shown in Figure 12c. The results indicate that inhomogeneous exposure of IWMDs to active Qi-A13 devices was within 46% of the performance limit even under worst-case conditions in a torso phantom. A 10 cm distance between the phantom and the Qi-A13 board guaranteed a safety margin for induced voltage levels 50 times below the permissible limit.

## 4. Compilation of Recent Publications on RF Transceiver-Based COTS-Enabled IWMDs

Table 6 represents an assortment of OEM and vendor RF transceiver-based COTS components which have been used to manufacture miniaturized IWMDs presented in the literature. RF transceivers are the most popular COTS components in IWMDs compared to PMU, microcontrollers and AFE due to their widely accepted custom-built chip counterparts. Table 6 lists the important parameters, such as manufacturer, wireless standard, source of power and the IWMD operation range for each of these RF transceiver COTS components. In addition to the following suggestions, the information provided in Table 6 can assist researchers and designers with the selection of COTS components for IWMD development.

- Near-field communications (NFC) and UHF RFID standards are suitable choices for battery-free IWMDs which operate over a very short-range (and contact-based) wireless connectivity and power transfer. In comparison with battery-operated MICS and BLE-enabled devices (ranging between −70 dBm and −100 dBm), implantable RFID devices have very poor RF receiver sensitivity levels (ranging between −10 and −20 dBm). This limits the operating range of battery-free RFID devices to a range of a few cm.
- Battery-operated COTS IWMDs can achieve wireless connectivity at distances greater than 1 m. However, they must contend with larger form factors and require rechargeable energy storage devices to operate over the desired application’s lifetime.
- A solar-powered implant potentially has an unlimited energy source and unlimited operating range [73] subject to the availability of direct sunlight. Additionally, the size of the solar cells to be used for IWMD is a concern.
- BLE devices [73,96,97] are easy to integrate and can provide a comparatively larger range and better data rate. However, the power consumption of the BLE devices is significantly higher and needs frequent charging of the battery.

**Table 6:** List of COTS-enabled IWMDs.

Compilation of Recent Publications on RF Transceiver-Based COTS-Enabled IWMDs					
Ref	COTS Component	Manufacturer (Vendor)	Wireless Standard	Power Source	Operating Range
[71]	SL900A RFID Sensory Tag	Austria Microsystems	UHF RFID	Battery-free	60 cm
[98]	EM4325 RFID Sensory Tag	EM Microelectronics	UHF RFID	WPT: 6.78 MHz	Not mentioned
[99]	MONZA-4 RFID Chip	IMPINJ	UHF RFID	Battery-free	≈35 cm
[100]	MONZA-4 RFID Die	IMPINJ	UHF RFID	Battery-free	≈35 cm
[101]	G2X RFID Chip	NXP	UHF RFID	Battery-free	20 cm
[102]	Alien Higgs-4 RFID Chip	Alien Technology	UHF RFID	Battery-free	10 cm
[103]	Alien Higgs-4 RFID Chip	Alien Technology	UHF RFID	Battery-free	Not mentioned
[104]	Alien Higgs-4 RFID Chip	Alien Technology	UHF RFID	Battery-free	Not mentioned
[92]	CC430F5137 SoC, PIC12LF1552	TI, Microchip	433 MHz NFC RFID	WPT: 6.67 MHz, Rechargeable battery	10 cm 8.8 cm
[105]	USRP B210 SDR	Ettus Research	UHF RFID	WPT: 13.56 MHz	5 mm
[47]	ZL70101	Microsemi	MICS 402 MHz	WPT 13.56 MHz	2 m
[106]	ZL70102	Microsemi	MICS 402 MHz	WPT TI: BQ51013	5 mm
[107]	ZL70101	Microsemi	Implant 2.45 GHz WuR: 420 MHz	WPT: 13.56 MHz	≈2 m
[108]	ZL70123	Microsemi	MICS 402 MHz WuR: 420 MHz	600 mAh Li-ion	≈2.5 m
[75]	ZL70102	Microsemi	MICS 402 MHz WuR: 2.45 GHz	WPT TI: BQ51013	1.98 m
[73]	RFD77101	SIMBLEE	BLE: 2.45 GHz	7 mAh rechargeable battery (VL1220) with solar panel	N/A
[96]	CC2640	TI	BLE: 2.45 GHz	Battery	Not mentioned
[97]	nRF51822	NORDIC	BLE: 2.45 GHz	Rechargeable Li-ion, WPT: 1.056 MHz	10 mm

## 5. Conclusions

This review highlights the versatility with which COTS components can be used for the development of IWMDs. Advances in material technologies and the development of novel micro- and nano-scale devices have been fundamental in the innovation of low-power miniaturized COTS components. Automated high-yield manufacturing processes have engendered the ubiquity of COTS used in the development of IWMDs. COTS components, particularly fully-integrated SoC modules, minimize BoM component count and associated time and costs for large-volume manufacture. Most manufacturers of COTS components provide hardware and software development tools and support resources throughout the application development process. Compared to a custom-built ASIC, the COTS components can significantly reduce the design cycle of an IWMD. Furthermore, the manufacturing cost of a single custom-built IC is extravagantly high.

COTS-enabled IWMDs play a fundamental role in healthcare networks, providing remote patient diagnostics and drug delivery services. In response to novel global epidemics such as COVID-19, the rapid development and deployment of high-quality first responder test equipment are crucial, where COTS components can offer robustness, reliability and significantly accelerate the development cycle for IWMDs. However, there are number of challenges associated with the utilization of COTS components for IWMDs. Current COTS components available on the market provide less opportunity for customization. Therefore, in many instances, it is difficult to find a COTS component that completely matches the requirements of a specific application. Furthermore, there are limited manufacturers of these COTS components, which can cause a monopoly during any pandemic situation and significantly increase costs. Higher demand from other industries can also limit the supply of a particular COTS component for IWMDs.

Although the majority of COTS components used to develop IWMDs are not designed for medical applications, research-generated concepts have demonstrated how versatile COTS components are. Commonly used ISM license-free wireless connectivity standards RIFD, BLE and MICS provide additional support for the development of IWMDs for better functionality, low cost and faster yield. Therefore, COTS-enabled devices offer promising research and manufacturing support for IWMDs as an alternative to custom building ASICs. Additionally, there are many promising opportunities for COTS components for IWMDs in the future. Modern programmable system on chip (PSoC) and field programmable gate array (FPGA) COTS devices provide designers the opportunity to customize IWMDs as per their requirements. Furthermore, many manufacturers are focusing on the production of significantly smaller form factor and low-power COTS components which can considerably reduce the size of the IWMDs and the power requirements, respectively. Manufacturers are also working to produce more flexible COTS sensors which can easily fit in critically curved body parts inside humans for IWMDs. The wireless aspects of the COTS-enabled IWMDs are also improving, as there are multiple new components emerging with higher bandwidth, distance requirements and significantly high-efficiency power supply.

## Figures and Tables

**Table 1:** Legacy of initial custom-built IWMDs.

Year	Application	Reference
1948	Radio Inductograph	[14]
1957	pH-Endoradiosonde	[15]
1962	Telemetry pill	[16]
1967	Electrocardiogram (ECG), electroencephalogram (EEG), wireless biotelemetry	[17]

**Table 2:** Legacy of commercial COTS-enabled IWMDs.

Year	Application	Reference
1966	A radio-telemetry device for monitoring temperature	[18]
1970	Implantable bio-telemetry systems	[19]
1999	Injectable electronic identification, monitoring and stimulation systems	[20]
2000	Advances in wireless telemedicine	[13]
2002	Wireless network for emergency medical care	[21]
2011	Wireless and implantable electrocorticogram recording system	[22]
2018	Wireless telemetry ingestible capsule	[23]
2021	Wireless monitoring of local deep infection	[24]

**Table 3:** COTS DRAs for miniaturized IWMDs.

	868 MHz DRAs	
Reference	Gain (dBi)	Size
[41]	−0.5	5 mm× 3 mm × 0.5 mm
[42]	−1	7 mm × 2 mm × 1.2 mm
[43]	+0.7	10 mm × 3 mm × 2 mm
[44]	+1.73	15.5 mm × 10.5 mm × 1.2 mm
[45]	−0.7	11 mm × 5.1 mm × 1.5 mm
[46]	1.5	9 mm × 3 mm × 0.63 mm

**Table 4:** COTS wireless transceivers.

Parameters	ZL70101	CC2541	Ucode7
Manufacturer	Microsemi	TI	NXP
Standard	*** MICS	** BLE 4.0	* UHF RFID
Frequency	402 to 405 MHz	2.4 GHz	860 to 960 MHz
Transmit current (mA)	5	18	Battery-free
Distance (m) free space	1000	100	15
Form factor	7 mm × 7 mm × 0.9 mm	6 mm × 6 mm × 1 mm	1.5 mm × 1 mm × 0.5 mm
Application	ECG	ECoG, EEG, GERD	Tracking
References	[47,50,51]	[22,52,53]	[54]

*** MICS: medical implant communication service. ** BLE: Bluetooth low energy. * UHF RFID: ultra-high-frequency radio frequency identification.

**Table 5:** COTS-enabled implantable neural interface.

	Bill of Materials		
VENDOR	Part Number	Description	Block
Intan Tech	RHD2164	64 CH. Chip	Recorder
Analog Devices	AD5863	16-bit DAC	Stimulus
	ADG506A	Multiplexer	
	LT1638	Opamp	
N/A	N/A	1 Zener Diode	Power Supply
	N/A	5 Capacitors	
	N/A	4 Diodes	
Lattice Semi	LCMX03LF-1300E	FPGA	Radio/MCU

## References

[1] John O. (2020). Maintaining and sustaining a telehealth-based ecosystem. Fundamentals of Telemedicine and Telehealth.

[2] (2010). World Health Organization Telemedicine: Opportunities and Developments in Member States: Report on the Second Global Survey on eHealth. https://apps.who.int/iris/bitstream/handle/10665/44497/9789241564144_eng.pdf?sequence=1&isAllowed=y.

[3] Kaushik A., Mujawar M.A. (2018). Point of Care Sensing Devices: Better Care for Everyone. Sensors.

[4] Ghafar-Zadeh E. (2015). Wireless integrated biosensors for point-of-care diagnostic applications. Sensors.

[5] Trautner B.W., Darouiche R.O. (2004). Catheter-associated infections: Pathogenesis affects prevention. Arch. Intern. Med..

[6] Johnson B.C., Gambini S., Izyumin I., Moin A., Zhou A., Alexandrov G., Santacruz S.R., Rabaey J.M., Carmena J.M., Muller R. An implanTable 700μW 64-channel neuromodulation IC for simultaneous recording and stimulation with rapid artifact recovery. Proceedings of the 2017 Symposium on VLSI Circuits.

[7] Bhamra H., Tsai J.W., Huang Y.W., Yuan Q., Shah J.V., Irazoqui P. (2017). A Subcubic Millimeter Wireless Implantable Intraocular Pressure Monitor Microsystem. IEEE Trans. Biomed. Circ. Syst..

[8] Chow E.Y., Chlebowski A.L., Chakraborty S., Chappell W.J., Irazoqui P.P. (2010). Fully wireless implantable cardiovascular pressure monitor integrated with a medical stent. IEEE Trans. Biomed. Eng..

[9] Charthad J., Chang T.C., Liu Z., Sawaby A., Weber M.J., Baker S., Gore F., Felt S.A., Arbabian A. (2018). A mm-Sized wireless implantable device for electrical stimulation of peripheral nerves. IEEE Trans. Biomed. Circuits Syst..

[10] Bisht R., Mandal A., Mitra A.K. (2017). Micro-and Nanotechnology-Based Implantable Devices and Bionics. Emerging Nanotechnologies for Diagnostics, Drug Delivery and Medical Devices.

[11] Meng E., Hoang T. (2012). Micro- and nano-fabricated implantable drug-delivery systems. Ther. Deliv..

[12] Kwong D.-L. (2011). Bringing the Benefits of Moore’s Law to Medicine.

[13] Kazmi A., Arslan B.R., Tulgar M., Pedersen P.C., Pahlavan K., Cengiz B., Arslan A. Advances in wireless telemedicine: Improvements and challenges facing wireless ultrasound. Proceedings of the 2000 IEEE EMBS International Conference on Information Technology Applications in Biomedicine, ITAB-ITIS.

[14] Fuller J.L., Gordon T.M. (1948). The Radio Inductograph—A Device for Recording Physiological Activity in Unrestrained Animals. Science.

[15] Jacobson B., Mackay R.S. (1957). A pH-endoradiosonde. Lancet.

[16] Watson B.W., Ross B., Kay A.W. (1962). Telemetering from within the body using a pressure-sensitive radio pill. Gut.

[17] Ko W.H., Neuman M.R. (1967). Implant Biotelemetry and Microelectronics. Science.

[18] Pipes E.W. (1966). Miniaturuzed Radiotelemetry Device for Monitoring Temperatures.

[19] Fryer T. (1970). Implantable Biotelemetry Systems: A Report.

[20] Troyk P.R. (1999). Injectable electronic identification, monitoring, and stimulation systems. Annu. Rev. Biomed. Eng..

[21] Fulford-Jones T., Welsh M., Moulton S., Malan D. CodeBlue: An Ad Hoc Sensor Network Infrastructure for Emergency Medical Care. Proceedings of the International Workshop on Wearable and Implantable Body Sensor Networks.

[22] Xie K., Zhang S., Dong S., Li S., Yu C., Xu K., Chen W., Guo W., Luo J., Wu Z. (2017). Portable wireless electrocorticography system with a flexible microelectrodes array for epilepsy treatment. Sci. Rep..

[23] Faerber J., Cummins G., Pavuluri S.K., Record P., Rodriguez A.R.A., Lay H.S., McPhillips R., Cox B.F., Connor C., Gregson R. (2017). In Vivo Characterization of a Wireless Telemetry Module for a Capsule Endoscopy System Utilizing a Conformal Antenna. IEEE Trans. Biomed. Circ. Syst..

[24] Avaltroni P., Nappi S., Marrocco G., Member S. (2021). Antennifying Orthopedic Bone-Plate Fixtures for the Wireless Monitoring of Local Deep Infections. IEEE Sens. J..

[25] Cohen T. (2004). Medical and information technologies converge. IEEE Eng. Med. Biol. Mag..

[26] Mahfouz M.R., Kuhn M.J., To G. Wireless medical devices: A review of current research and commercial systems. Proceedings of the IEEE Topical Conference on Biomedical Wireless Technologies, Networks, and Sensing Systems.

[27] Elayan H., Shubair R.M., Kiourti A. Wireless sensors for medical applications: Current status and future challenges. Proceedings of the 11th European Conference on Antennas and Propagation (EUCAP).

[28] WHO (2010). A comprehensive guide to the Global Medical Device Nomenclature. https://www.who.int/medical_devices/innovation/GMDN_Agency_User_Guide_v120810.pdf.

[29] (2022). FDA Medical Device Databases. https://www.fda.gov/medical-devices/device-advice-comprehensive-regulatory-assistance/medical-device-databases.

[30] (1993). US Airforce Material Command: COTS Guide. https://nepp.nasa.gov/DocUploads/1219C61B-7337-48C4-8760E6456F861839/COTSguide.pdf.

[31] (2022). Texas Instruments INC Components for Space, Avionics and Defense. www.ti.com/SpaceAvionicsDefense.

[32] IEC/IEEE 62704-1 (2017). IEC/IEEE International Standard—Determining the Peak Spatial-Average Specific Absorption Rate (SAR) in the Human Body from Wireless Communications Devices, 30 MHz to 6 GHz—Part 1: General Requirements for Using the Finite-differ. https://standards.ieee.org/ieee/62704-1/5747.

[33] (2012). Health Effects from Radiofrequency Electromagnetic Fields Report of the Independent Advisory Group on Non-Ionising Radiation. https://www.gov.uk/government/publications/radiofrequency-electromagnetic-fields-health-effects.

[34] (2015). Scenihr Opinion on Potential health effects of exposure to electromagnetic fields (EMF). EU Sci. Comm..

[35] Madjar H.M. (2016). Human radio frequency exposure limits: An update of reference levels in Europe, USA, Canada, China, Japan and Korea. IEEE Int. Symp. Electromagn. Compat..

[36] ICNIRP (2020). Guidelines for Limiting Exposure to Electromagnetic Fields (100 kHz to 300 GHz). Health Phys..

[37] Bocan K.N., Mickle M.H., Sejdic E. (2017). Multi-Disciplinary Challenges in Tissue Modeling for Wireless Electromagnetic Powering: A Review. IEEE Sens. J..

[38] Agarwal A., Shapero A., Rodger D., Humayun M., Tai Y.C., Emami A. A wireless, low-drift, implantable intraocular pressure sensor with parylene-on-oil encapsulation. Proceedings of the 2018 IEEE Custom Integrated Circuits Conference (CICC).

[39] LPS25H (2018). MEMS Pressure Sensor: 260-1260 hPa Absolute Digital Output Barometer—STMicroelectronics. https://www.st.com/en/mems-and-sensors/lps25h.html.

[40] George J., Compagno T., Rodgers K., Waldron F., Barrett J. (2015). Reliability of Plastic-Encapsulated Electronic Components in Supersaturated Steam Environments. IEEE Trans. Components Packag. Manuf. Technol..

[41] (2021). 868 MHz Embedded Ceramic Loop Antenna for ISM/Lora/LPWAN/Sigfox. https://www.taoglas.com/product/530-5-ila-08-868mhz-embedded-ceramic-loop-antenna-ismloralpwansigfox.

[42] Johanson Technology 868 MHz Antenna for Small Form Factor Applications. http://www.farnell.com/datasheets/2280483.pdf.

[43] 868/915 MHz (2016). KIRBII, ISM SMD antenna—Antenova. http://antenova.com/wp-content/uploads/2015/11/Kirbii-A10472-PS-5.1.pdf?__hstc=176912634.486337ce428aefc1c912f83df14de3df.1626276596230.1626276596230.1626276596230.1&__hssc=176912634.1.1626276596231&__hsfp=2875711223&hsCtaTracking=459d335a-4319-4a90-bd00.

[44] (2013). Surface Mount Ceramic Chip Antennas for 868 MHz. https://media.digikey.com/pdf/DataSheets/VishayVitramon/VJ5601M868MXBSR.pdf.

[45] (2021). WE-MCA Multilayer Chip Antenna. https://www.we-online.com/catalog/datasheet/7488910092.pdf.

[46] Cheng K. (2020). Molex 2081420001-ISM 868/915 MHz Ceramic Antenna. https://www.molex.com/pdm_docs/ps/2081420001-PS.pdf.

[47] Charvet G., Foerster M., Filipe S., Porcherot J., Beche J.F., Guillemaud R., Audebert P., Regis G., Zongo B., Robinet S. WIMAGINE: A wireless, low power, 64-channel ECoG recording platform for implantable BCI applications. Proceedings of the 5th International IEEE/EMBS Conference on Neural Engineering.

[48] (2013). 4-GHz BluetoothTM Low Energy and Proprietary System-on-Chip. https://www.ti.com/lit/ds/symlink/cc2541.pdf?ts=1640439778669&ref_url=https%253A%252F%252Fwww.google.com%252F.

[49] (2019). NXP Semiconductors UCODE® 7/7m. https://www.nxp.com/products/rfid-nfc/ucode-rain-rfid-uhf/ucode-7-7m:SL3S1204.

[50] Higgins H. (2009). Two way wireless communication with implanted sensors and therapeutic devices. IET Semin. Dig..

[51] Higgins H. (2007). Body implant communication—Is it a reality?. IET Semin. Dig..

[52] Ghomashchi A., Zheng Z., Majaj N., Trumpis M., Kiorpes L., Viventi J. A low-cost, open-source, wireless electrophysiology system. Proceedings of the 2014 36th Annual International Conference of the IEEE Engineering in Medicine and Biology Society.

[53] Sun X.C., Tao W.J., Zhu C.Q., Zhao L.L., Wang M., Lu X.Y., Wang Z.G., Fan Z.N. System design and experimental research of lower esophageal sphincter stimulator for treatment of gastroesophageal reflux disease. Proceedings of the IEEE Engineering in Medicine and Biology Society (EMBC).

[54] Gottardi D., Wienstroer V., Kronberger R. Animal Skin Phantom for RFID UHF Transponder Development. Proceedings of the 2018 International Symposium on Antennas and Propagation (ISAP).

[55] (2015). ZL70101 Medical Implantable RF Transceiver Features. https://www.microsemi.com/document-portal/doc_view/127877-zl70101-1645-19-fullds-datasheet.

[56] Silva N.M., Santos P.M., Ferreira J.A.F., Soares Dos Santos M.P., Ramos A., Simões J.A.O., Reis M.J.C.S., Morais R. (2013). Power management architecture for smart hip prostheses comprising multiple energy harvesting systems. Sens. Actuators A Phys..

[57] Pederson D.J., Quinkert C.J., Arafat M.A., Somann J.P., Williams J.D., Bercich R.A., Wang Z., Albors G.O., Jefferys J.G.R., Irazoqui P.P. (2019). The Bionode. ACM Trans. Embed. Comput. Syst..

[58] Serra P.A., Rocchitta G., Bazzu G., Manca A., Puggioni G.M., Lowry J.P., O’Neill R.D. (2007). Design and construction of a low cost single-supply embedded telemetry system for amperometric biosensor applications. Sens. Actuators B.

[59] (2004). PIC12F683 8-Pin flash-based, 8-Bit CMOS Microcontrollers with NanoWatt Technology. http://ww1.microchip.com/downloads/en/devicedoc/41211b.pdf.

[60] Arshak K., Lyons G., Cavanagh L., Clifford S. (2003). Front-end signal conditioning used for resistance-based sensors in electronic nose systems: A review. Sens. Rev..

[61] Horn G.V.D., Huijsing J.H. (1997). Integrated Smart Sensor Calibration. Analog. Integr. Circ. Sign. Process..

[62] Wilson A.D., Baietto M. (2009). Applications and Advances in Electronic-Nose Technologies. Sensors.

[63] Lee J.H., Seo D.W. (2019). Development of ECG Monitoring System and Implantable Device with Wireless Charging. Micromachines.

[64] Bilodeau G., Gagnon-Turcotte G., Gagnon L.L., Keramidis I., Timofeev I., De Koninck Y., Ethier C., Gosselin B. (2021). A Wireless Electro-Optic Platform for Multimodal Electrophysiology and Optogenetics in Freely Moving Rodents. Front. Neurosci..

[65] Ukkonen L., Sydänheimo L., Ma S., Björninen T. Backscattering-based wireless communication and power transfer to small biomedical implants. Proceedings of the Microfluidics, BioMEMS, and Medical Microsystems XVIII.

[66] Großmann S., Ott R., Kosub R., Schmitz K.-P., Siewert S., Schmidt W., Grabow N. (2019). Numerical investigation of stent designs for wireless access to integrated sensors. Curr. Dir. Biomed. Eng..

[67] Shapero A., Agarwal A., Martinez J.C., Emami A., Humayun M.S., Tai Y.C. Wireless Implantable Intraocular Pressure Sensor with Parylene-Oil-Encapsulation and Forward-Angled RF Coil. Proceedings of the IEEE 32nd International Conference on Micro Electro Mechanical Systems.

[68] Becker J., Pellhammer D., Preißner P., Glöggler J.C., Lapatki B.G., Ortmanns M. An implant for wireless in situ measurement of lip pressure with 12 sensors. Proceedings of the 2017 IEEE Biomedical Circuits and Systems Conference.

[69] SL900A EPC Sensor Tag and Data Logger IC. 2018. https://ams.com/sl900a.

[70] Di Trocchio L., Carucci C., Sindhu K.R., Morel C., Lachaud J.L., Bichon S., Gounel S., Mano N., Boiziau C., Dejous C. (2021). Wireless in Vivo Biofuel Cell Monitoring. IEEE J. Electromagn. RF Microw. Med. Biol..

[71] Vena A., Sorli B., Charlot B., Naudi S. An RFID-based implant for identification and pressure sensing of orthopedic prosthesis. Proceedings of the 1st URSI Atlantic Radio Science Conference (URSI AT-RASC); Institute of Electrical and Electronics Engineers (IEEE).

[72] Lazaro M., Lazaro A., Villarino R. (2020). Feasibility of backscatter communication using LoRAWAN signals for deep implanted devices and wearable applications. Sensors.

[73] Wu T., Redouté J.M., Yuce M.R. (2018). A Wireless Implantable Sensor Design With Subcutaneous Energy Harvesting for Long-Term IoT Healthcare Applications. IEEE Access.

[74] Bluetooth S. (2015). SimbleeTM RFD77101 IoT for Connecting Everyone and Everything. https://cdn.sparkfun.com/datasheets/IoT/Simblee%20RFD77101%20Datasheet%20v1.0.pdf.

[75] Shon A., Chu J.-U., Jung J., Kim H., Youn I. (2017). An Implantable Wireless Neural Interface System for Simultaneous Recording and Stimulation of Peripheral Nerve with a Single Cuff Electrode. Sensors.

[76] Bentler C., Stieglitz T. Building wireless implantable neural interfaces within weeks for neuroscientists. Proceedings of the 39th Annual International Conference of the IEEE Engineering in Medicine and Biology Society (EMBC).

[77] Bock D.C., Marschilok A.C., Takeuchi K.J., Takeuchi E.S. (2012). Batteries used to Power Implantable Biomedical Devices. Electrochim. Acta.

[78] Takeuchi E.S., Leising R.A. (2002). Lithium Batteries for Biomedical Applications. MRS Bull..

[79] (2016). Qualliion Medical Batteries: Powering Life. https://www.enersys.com/en/products/batteries/quallion/quallion-lithium.

[80] Young D.J., Cong P., Suster M.A., Damaser M. (2015). Implantable wireless battery recharging system for bladder pressure chronic monitoring. Lab Chip.

[81] J. Macsalka. Micro3—QL0003B Rechargeable Lithium Ion Battery. https://medical.enersys.com/products.

[82] Bhatia N., El-Chami M. (2018). Leadless pacemakers: A contemporary review. J. Geriatr. Cardiol..

[83] Liu G., Mao L., Chen L., Xie S. (2014). Locatable-Body Temperature Monitoring Based on Semi-Active UHF RFID Tags. Sensors.

[84] Fernández-Salmerón J., Rivadeneyra A., Martínez-Martí F., Capitán-Vallvey L.F., Palma A.J., Carvajal M.A. (2015). Passive UHF RFID Tag with Multiple Sensing Capabilities. Sensors.

[85] Rennane A., Fonseca N., Abdelnour A., Benmahmoud F., Kaddour D., Touhami R., Tedjini S. Passive UHF RFID Sensor Tag for Pressure and Temperature Conditions Monitoring. Proceedings of the 2nd URSI Atlantic Radio Science Meeting (AT-RASC).

[86] De Donno D., Catarinucci L., Tarricone L. (2014). RAMSES: RFID augmented module for smart environmental sensing. IEEE Trans. Instrum. Meas..

[87] Jung J.Y., Kim H., Lee H.S., Yeom K.W. An UHF RFID tag with long read range. Proceedings of the 2009 European Microwave Conference (EuMC).

[88] Khan S.R., Desmulliez M.P.Y. (2019). Implementation of a Dual Wireless Power Transfer and Rotation Monitoring System for Prosthetic Hands. IEEE Access.

[89] Khan S.R., Desmulliez M.P.Y. (2019). Towards a Miniaturized 3D Receiver WPT System for Capsule Endoscopy. Micromachines.

[90] Biswas D.K., Tasneem N.T., Mahbub I. Optimization of Miniaturized Wireless Power Transfer System to Maximize Efficiency for Implantable Biomedical Devices. Proceedings of the IEEE Texas Symposium on Wireless and Microwave Circuits and Systems (WMCS).

[91] Ahmadi M.M., Sarbandi-Farahani M. (2020). A Class-E Power and Data Transmitter with On–Off Keying Data Modulation for Wireless Power and Data Transmission to Medical Implants. Circ. Syst. Signal Process..

[92] Bakula M., Pelgrims P., Puers R. (2016). Wireless powering and communication for implants, based on a Royer oscillator with radio and near-field links. Sens. Actuators A.

[93] Seckler T., Jagielski K., Stunder D. (2015). Assessment of Electromagnetic Interference with Active Cardiovascular Implantable Electronic Devices (CIEDs) Caused by the Qi A13 Design Wireless Charging Board. Int. J. Environ. Res. Public Health.

[94] (2018). Wireless Power Consortium. https://www.wirelesspowerconsortium.com.

[95] WCT1001A/WCT1003A (2014). Automotive A13 Wireless Charging Application User’s Guide. https://www.nxp.com/docs/en/user-guide/WCT100XAWCAUG_V3.5.pdf.

[96] Zhou L., Chen X., Li Y., Li J. Bluetooth low energy 4. 0-based communication method for implants. Proceedings of the 10th International Congress on Image and Signal Processing, BioMedical Engineering and Informatics CISP-BMEI.

[97] Su Y., Routhu S., Moon K.S., Lee S.Q., Youm W., Ozturk Y. (2016). A Wireless 32-Channel Implantable Bidirectional Brain Machine Interface. Sensors.

[98] Kracek J., Švanda M., Mazanek M., Machac J. (2016). Implantable Semi-Active UHF RFID Tag with Inductive Wireless Power Transfer. IEEE Antennas Wirel. Propag. Lett..

[99] Lodato R., Marrocco G. (2016). Close Integration of a UHF-RFID Transponder into a Limb Prosthesis for Tracking and Sensing. IEEE Sens. J..

[100] Lodato R., Lopresto V., Pinto R., Marrocco G. (2014). Numerical and experimental characterization of through-the-body UHF-RFID links for passive tags implanted into human limbs. IEEE Trans. Antennas Propag..

[101] Occhiuzzi C., Contri G., Marrocco G. (2012). Design of implanted RFID tags for passive sensing of human body: The STENTag. IEEE Trans. Antennas Propag..

[102] Garcia-Miquel A., Medina-Rodriguez B., Vidal N., Ramos F.M., Roca E., Lopez-Villegas J.M. Design and characterization of a miniaturized implantable UHF RFID tag based on LTCC technology. Proceedings of the 11th European Conference on Antennas and Propagation (EUCAP).

[103] Xu J., Sato H., Motoyoshi M., Suematu N., Kanetaka H., Yasui K., Chen Q. Development of Denture Implanted RFID Tag Antennas. Proceedings of the IEEE International Workshop on Electromagnetics: Applications and Student Innovation Competition (iWEM).

[104] Rajagopalan H., Rahmat-Samii Y. Ingestible RFID bio-capsule tag design for medical monitoring. Proceedings of the IEEE Antennas and Propagation Society International Symposium.

[105] Kampianakis E., Sharma A., Arenas J., Reynolds M.S. (2017). A dual-band wireless power transfer and backscatter communication approach for implantable neuroprosthetic devices. IEEE J. Radio Freq. Identif..

[106] Rotermund D., Pistor J., Hoeffmann J., Schellenberg T., Boll D., Tolstosheeva E., Gauck D., Stemmann H., Peters-Drolshagen D., Kreiter A.K. (2017). Open Hardware: Towards a Fully-Wireless Sub-Cranial Neuro-Implant for Measuring Electrocorticography Signals. bioRxiv.

[107] Fang Q., Lee S.Y., Permana H., Ghorbani K., Cosic I. (2011). Developing a wireless implantable body sensor network in MICS band. IEEE Trans. Inf. Technol. Biomed..

[108] Tantin A., Letourneau A., Zgaren M., Hached S., Clausen I., Sawan M. Implantable MICS-based wireless solution for bladder pressure monitoring. Proceedings of the 2017 IEEE Biomedical Circuits and Systems Conference (BioCAS).

